# The relationship between distributed leadership and teacher innovativeness: Mediating roles of teacher autonomy and professional collaboration

**DOI:** 10.3389/fpsyg.2022.948152

**Published:** 2022-07-27

**Authors:** Qi Lin

**Affiliations:** Faculty of Education, East China Normal University, Shanghai, China

**Keywords:** distributed leadership, teacher innovativeness, teacher autonomy, professional collaboration, TALIS

## Abstract

Principals’ distributed leadership plays a critical role in teacher innovativeness; however, research evidence regarding the relationship between them is limited. This study aims at examining the effect of distributed leadership on teacher innovativeness as well as the mediating roles of teacher autonomy and professional collaboration. Using the data of 132,376 teachers derived from the 2018 Teaching and Learning International Survey (TALIS), the study applied a structural equation model (SEM) for analysis. The results revealed that distributed leadership had positive direct effects on teacher innovativeness, teacher autonomy, and professional collaboration. Meanwhile, teacher autonomy and professional collaboration significantly mediated the effect of distributed leadership on teacher innovativeness, respectively. Practical implications are discussed, school leaders are expected to adopt distributed leadership style and establish a supportive school environment, and individual teachers are supposed to cultivate a culture of collectivism and make effective use of autonomy in their teaching innovation.

## Introduction

Innovation in education is essential for generating beneficial changes for school improvement and long-term sustainability (Serdyukov, 2017). Unquestionably, teacher inventiveness, which is conceptualized as teacher responsiveness, openness, and readiness to adopt reform and change (McGeown, 1980; Amels et al., 2020), is crucial to the implementation of educational innovations in classrooms and schools (Kern and Graber, 2018; Brown et al., 2020). It is viewed not only as a process of producing, advocating, and achieving change but also as teachers’ capacity to integrate these innovations into personal and communal practices. Out of this importance, teacher innovativeness has been a heated topic in education reforms and school development globally, as well as the rising academic interest (Thurlings et al., 2015). And there is no doubt that exploring the predictors of teacher innovativeness shows great significance.

Research have revealed that teacher inventiveness is influenced by a combination of organizational and individual factors. Accordingly, a small but rising number of theoretical and empirical studies have identified significant predictors that may affect teacher inventiveness, such as distributed leadership. Distributed leadership is considered one of the most important organizational predictors in the school. As Spillane et al. (2004) stated, educational innovation requires creative organizational structures and decentralized leadership. Several studies have also demonstrated that principals’ distributed leadership may considerably enhance the inventive capacities and performance of their staff (Leithwood et al., 2009).

Nevertheless, according to previous studies, there are still two distinct ways in which teachers inspire innovation: individually and collectively (Torres, 2019; Liu et al., 2021b). From this viewpoint, teacher autonomy, which is frequently viewed as individualism in the teaching profession, is essential to teacher inventiveness due to its empowerment of individuals. Professional collaboration, on the contrary, has traditionally been recognized as collectivism in the work of teachers and is also acknowledged as a critical way to foster teachers’ networking and generate collective innovativeness. In addition, studies have also revealed that teacher autonomy and professional collaboration might be influenced by principals’ distributed leadership (Nguyen et al., 2021), indicating that teacher autonomy and professional collaboration may serve as mediators between distributed leadership and teacher innovativeness. However, the relationships among these variables have not yet been thoroughly examined.

Therefore, the current study aims at using the large-scale teacher data derived from the 2018 Teaching and Learning International Survey (TALIS) to answer two research questions: (1) How does distributed leadership directly affect teacher innovativeness? and (2) do teacher autonomy and professional collaboration mediate the effect of distributed leadership on teacher innovativeness? This study contributes to an international knowledge of the impact of distributed leadership on teacher innovativeness and the functions of teacher autonomy and professional collaboration as mediators, aiming at providing generalizable evidence for countries and economies from different contexts. In another word, this study will offer evidence-based ideas for promoting teacher innovativeness that supports continuous school improvement from a global perspective.

## Literature review

### Teacher innovativeness

It is well accepted that schools function in challenging and constantly changing contexts. Therefore, teachers are expected to continually maintain and improve their personal innovativeness to preserve the quality of education (Serdyukov, 2017). Innovativeness is a necessary trait for all individuals since it relates to the improvement and design of a product or service, and it is widely accepted in numerous fields (Lam et al., 2010). Teacher innovativeness is defined as teachers’ openness, acceptance, and internalization of creative ideas, as well as their ongoing engagement in innovation-related professional practices (Cohen-Vogel et al., 2016). It is regarded as the degree of teachers’ capacity and ability to change and is also a vital contributor to enhanced teacher performance, student academic achievement, and organizational development (McGeown, 1980; Serdyukov, 2017). Moreover, this concept was determined as a complex construct by McGeown (1980) comprising values, attitudes, adoption, commitment, engagement, and behaviors. Specifically, teacher innovativeness necessitates the development of new pedagogical or evaluative methods or strategies intended to improve the teaching and learning quality, and these innovative concepts might originate from the educational departments, the school administrators, or the teachers themselves.

According to the Person-Environment Fit theory, teacher innovativeness may be affected by a multi-layer of factors. Certain styles of leadership and school climate were found to favorably affect the innovative behavior of teaching staff (Sagnak, 2012). Studies suggested that distributed leadership of school leaders played a crucial role in fostering innovative processes and guiding these initiatives (Brown et al., 2020). It enables teachers to utilize their knowledge, passion, and imagination, which is vital to the success of educational improvements (Fullan, 2007; Seashore Louis and Lee, 2016; Buske, 2018). As a result, teachers are engaged in decisions on which innovations to embrace and how to implement them, such as analyzing how new practices may be utilized to improve teaching and how to continue to use/refine teaching techniques over time (Hallinger and Heck, 2010; Bush and Glover, 2014). For example, a case study in England revealed that if principals hold distributed leadership approaches, teacher professional learning network’s innovation would be positively supported (Brown et al., 2020). Therefore, it can be hypothesized that principals’ distributed leadership may have a direct effect on teacher innovativeness.

Because teachers’ professional environment is molded both individually and collectively, their perceptions of themselves as individuals and the faculty members as a community should be crucial to teachers’ innovation performance (Schechter and Tschannen-Moran, 2006). Therefore, in terms of innovativeness, both individual teachers and collective organizations may be important requirements. Following this hypothesis, recent studies have examined the individual preconditions of teachers, such as teacher autonomy, involved in innovation processes (Nguyen et al., 2021). At the same time, studies also highlighted the significance of the collective predictors to teacher innovative behaviors, particularly professional collaboration (Lanford et al., 2019). Through these analyses, teacher autonomy and professional collaboration have been identified as strong indicators of teacher innovativeness and openness to change, as well as significant determinants in innovation activities and educational quality improvement.

In sum, this study has identified three potential factors influencing teacher innovativeness based on the literature: distributed leadership, teacher autonomy, and professional collaboration. The subsequent sections are organized by these main factors.

### Distributed leadership

Distributed leadership is a helpful notion for comprehending how diverse organizational stakeholders, such as principals, teachers, students, and parents, practice leadership. Distributed leadership is widely known to facilitate an extended perspective of school leadership beyond the school principals’ activities. It enables researchers to view leadership as a dynamic interaction rather than a fixed functional manifestation (Gronn, 2002; Spillane and Healey, 2010). Tian et al. (2016) distinguished distributed leadership from other leadership concepts through a review of the literature. According to them, distributed leadership indicates that leadership in an organization is a reactive, participatory process that involves group-forming participants. This process has both ascending and descending hierarchical effects (Spillane et al., 2001). Its objective is to guide and support one another in the organization in order to accomplish the institution’s goals. From the standpoint of distributed leadership, multiple individuals can engage in leadership and management to accomplish the shared goals of a community (Leithwood et al., 2008). Moreover, this definition of leadership is distinct from others that link it with informal leaders, and in this way enriches the conception of leadership (Marks and Printy, 2003; Heck and Hallinger, 2009).

Over recent decades, a significant amount of educational studies have examined the impacts of distributed leadership on educational procedures and achievements (Brown et al., 2020). Notably, great emphasis has been made on distributed leadership’s influence on school effectiveness, teaching capacities, and success (Cerit, 2010), as well as its influence on teachers’ attitudes, expertise, and behaviors, including on teachers’ innovativeness (Buske, 2018). It was discovered that the principals’ leadership considerably and significantly encourages the teachers’ creativity (Liu et al., 2018). A recent study on successful school leadership highlighted the significance of enthusiastic support for teaching, open dialog, and the beneficial effect of school principals’ distributed leadership on fostering organizational culture and individual innovation (Daniëls et al., 2019).

In the meanwhile, Liu et al. (2021b) claimed that principals might also promote teacher autonomy and professional collaboration through distributed leadership. Teachers would be satisfied if they have autonomy or collaborate with peers in school under the distributed climate (Strong and Yoshida, 2014). Several empirical investigations have also confirmed that principals’ leadership style is crucial for determining the degree of teacher autonomy and collective activities (Luschei and Jeong, 2021). For instance, using teachers’ data from the 2013 TALIS, one study indicated that distributed leadership significantly impacted teachers’ professional learning community and cooperation practices (Bellibaş et al., 2021). Based on the literature reviewed above, we can assume that distributed leadership may, respectively, influence teacher innovativeness, teacher autonomy, and professional collaboration.

### Teacher autonomy

Worldwide emphasis has been placed on teacher autonomy as a critical factor supporting teachers’ practices in schools (Pearson and Hall, 1993; Pearson and Moomaw, 2006; Lepine, 2007; Benson, 2010). Gwaltney (2012) defined teacher autonomy as the extent to which teachers are provided with sufficient freedom, liberty, authority, and discretion to engage in organizing, choosing, and carrying out managerial, instructional, and socializing tasks in the class and school organization. The multi-dimensional characteristic of teacher autonomy has been widely acknowledged, and it can be primarily divided into two components. The first component is professional freedom, which refers to the degree of independence and control a teacher has over crucial classroom issues, such as lesson preparation, teaching materials, curriculum development, and student evaluation (Ingersoll and Merrill, 2011; Nguyen and Walkinshaw, 2018). The second component is school functioning capacity, which focuses on teachers’ capabilities to make decisions and function within context-specific restrictions, such as school culture and policy background (Benson, 2010). The current study defines teacher autonomy in terms of classroom instructional decision-making rather than school-level functioning.

Literature has detailed several elements that influence teachers’ perceptions of their autonomy, with an emphasis on organizational culture and school structure (Benson, 2010). Ingersoll and Merrill (2011) suggested that the institutional context of a school was a necessary requirement for achieving teacher autonomy. According to their research, teachers perceived more individual autonomy within a supportive working environment and distributed leadership (Talbert and McLaughlin, 1994; Wenner and Campbell, 2017). That is to say, the degree of school leaders’ control is closely related with teachers’ autonomy (Ingersoll and Smith, 2004).

In terms of the outcomes of teacher autonomy, it is recognized that teacher autonomy is one of the essential predictors of teachers’ perceptions of professionalism (Bauman, 2015) and their sense of independence (Rinehart et al., 1998). Moreover, it is related to teachers’ job satisfaction (Pearson and Moomaw, 2005; Wermke et al., 2019), work engagement (Skaalvik and Skaalvik, 2014), job commitment (Katzenmeyer and Moller, 2001), and turnover intention (Pearson and Hall, 1993). Furthermore, the degree of freedom to experiment and innovate in their classroom is also crucial to the achievement of school reforms and innovations. Buske (2018) argued that the autonomy of individuals in a school is critical for the effective implementation of school improvement, changes, and advances.

### Professional collaboration

Professional collaboration implies that teachers engage in lesson planning and problem-solving collectively, and share their knowledge, perspectives, and teaching strategies with one another to generate innovative instructional practices (Lomos et al., 2011). Moreover, it promotes the professional development of teaching staff. According to Goddard et al. (2007), the term “professional collaboration” can refer to a diverse assortment of activities carried out inside educational institutions. Specifically, the activity of collaboration is sometimes seen as a continuum, ranging from teachers working together on a one-time basis to those working together more intensively and frequently (Vangrieken et al., 2015). Through such a collective procedure, it is able to enhance the education quality provided to a single pupil, the whole classroom, and an entire school (Meirink et al., 2010).

It was shown that professional collaboration was a factor that positively correlated with teacher work engagement (Ronfeldt et al., 2015), job satisfaction (Heck and Hallinger, 2009), teacher self-efficacy (Torfing, 2019), and school academic performance (Thoonen et al., 2012). Studies have also discovered that teachers who have higher levels of collegiality, including the free exchange of ideas and resources, were more committed to their schools (Hausman and Goldring, 2001). In a study conducted in Turkey, Duyar et al. (2013) showed that cooperation between teachers, such as co-teaching, peer observation, and corporate dialog, increased work satisfaction among teachers.

Nevertheless, professional collaboration also demonstrates a predictive effect on school staffs’ innovativeness. Fullan (2016) highlighted that professional collaboration is vital for the success of school innovations. Generally, engaging in educational transformation demands a collegial workplace culture wherein teachers may share unique methods and offer peer support, strengthening their engagement with shared aims (Hausman and Goldring, 2001; Goddard et al., 2007). In turn, this sharing of responsibilities promotes the collaborative nature of work on innovations (Fullan, 2016). Thus, professional collaboration is distinguished by a vertical and horizontal multi-actor approach to innovation in which resources, expertise, and thoughts are communicated, leading to reciprocal progress (Duyar et al., 2013). However, an investigation of 473 Chinese EFL teachers found that teachers’ perceived peer support did not predict teacher innovations in the online teaching environment (Han et al., 2021). Although this finding was not discovered in an international context, it still provided the converse evidence to the existing literature from a country’s perspective. Therefore, the correlation between teacher collaboration and their innovativeness needs to be examined furtherly in this study.

Through the literature that has been reviewed above, we have formed and hypothesized the separate relationships regarding distributed leadership, teacher autonomy, professional collaboration, and teacher innovativeness. However, a conceptual framework still needs to be shaped to comprehensively and intuitively clarify the relationships between these four variables.

## Conceptual framework

**Figure 1:**
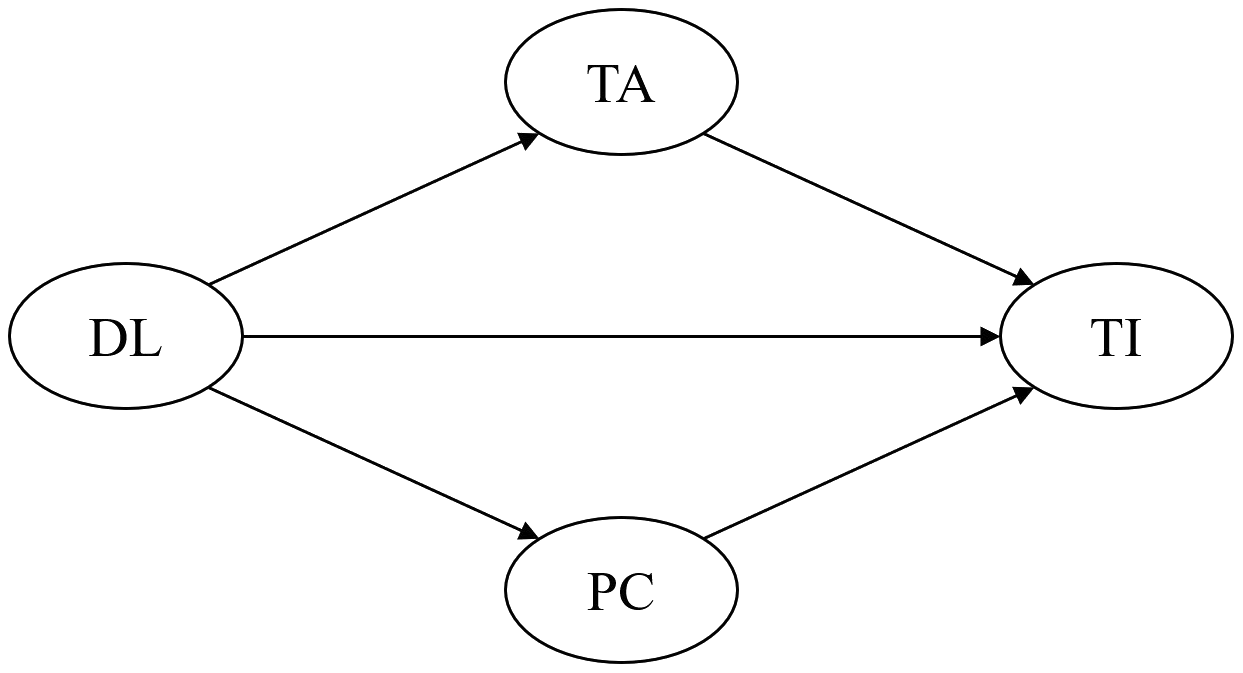
Conceptual framework. DL, Distributed leadership; TA, Teacher autonomy; PC, Professional collaboration; and TI, Teacher innovativeness.

Figure 1 illustrates the relationships between the variables of interest in this study, namely distributed leadership (DL), teacher autonomy (TA), professional collaboration (PC), and teacher innovativeness (TI).

In this conceptual framework, distributed leadership is the key independent variable. Its direct effects on teacher innovativeness, teacher autonomy, and professional collaboration are proposed based on the existing literature about the possible relationships among these factors. For example, studies indicated that distributed leadership not only impacted innovations that were successfully implemented (Buske, 2018) but also influenced teacher autonomy and professional collaboration, respectively (Moolenaar et al., 2010; Liu et al., 2021b). Besides this, teacher autonomy and professional collaboration are also considered predictors of teacher innovativeness. Evidence revealed that teacher autonomy and collaboration played significant roles in their innovativeness (McCharen et al., 2011).

Meanwhile, teacher autonomy and professional collaboration are posited as mediators between distributed leadership and teacher innovativeness for three reasons. Firstly, the impact of teacher autonomy and professional collaboration on teacher innovativeness can be linked to the effects of distributed leadership on teacher autonomy and collaboration logically. Secondly, several theoretical studies have implied that distributed leadership affected teacher innovativeness through the mediation of teacher autonomy and collaboration. For instance, when school members are strengthened with the autonomy and form of collaboration that comes with distributed leadership, they are better equipped to create and effectively scale up the application of innovations, as demonstrated by Kotter and Cohen (2014). Thirdly, the mediating effects of teacher autonomy and professional collaboration have been discovered between principals’ distributed leadership and teacher job satisfaction in some previous studies (Torres, 2019; Liu et al., 2021a), hinting that distributed leadership may also affect teacher innovativeness through teacher collaboration and autonomy.

## Methodology

### Data

This study applied a global database from the 2018 TALIS conducted by Organization for Economic Cooperation and Development (OECD). TALIS investigated teachers’ and principals’ daily practices, professional development, and workplace conditions in lower secondary education (ISCED 2). Cross-sectional data were collected in 48 countries and economies between September 2017 and November 2018 using standardized questionnaires. Although the main aim of TALIS is to generate globally comparable evidence for countries and economies, it seeks to provide important indicators and policy-relevant analysis of teachers and teaching from an international perspective based on the integral data (OECD, 2019a). Under this concern, together with the goal of this study, teacher data from participating countries and economies were grouped together for the analysis.

After excluding 15 countries/economies (Australia, Belgium, Bulgaria, France, Italy, New Zealand, Norway, Portugal, Shanghai (China), Singapore, Slovak Republic, Slovenia, Spain, Sweden, and Vietnam) which did not meet the acceptable values of reliability and validity of each construct (See the Analytical Approach and Preliminary Analysis sections) and deleting cases with missing values for the variables included in this study, the final sample for analysis was 132,376 teachers from 33 countries/economies. The sample included 90,030 females (68.0%) and 42,346 males (32.0%). Regarding teachers’ age, 11.5% were under 30 years, 29.8% were between 30 and 39 years, 31.1% were between 40 and 49 years, and 27.6% were above 50 years.

### Measures

This study adopted four variables, including one independent variable (distributed leadership), two mediating variables (teacher autonomy and professional collaboration), and one dependent variable (teacher innovativeness). All variables and their attached items were selected from the teacher questionnaire according to the TALIS technical report and analysis plan (OECD, 2019a,b).

*Distributed leadership* was a latent variable measured by a 4-point Likert scale, with 1 = strongly disagree, 2 = disagree, 3 = agree, and 4 = strongly agree. The scale consisted of five items aimed at investigating the degree of participation of various stakeholders in the school (e.g., “This school provides staff with opportunities to actively participate in school decisions”). Besides, it is worth noting that TALIS used the same items to measure distributed leadership both from teachers’ perspectives and principals’ perspectives. This study chose to use teachers’ perceived distributed leadership since it can represent the real work situation teachers are facing and is aligned with the focus of this study which concentrates on the relationship between teacher-level variables. Meanwhile, it can also help to maintain more valid cases for analysis.

As the mediating variable, *teacher autonomy* was an independent construct under teachers’ job satisfaction investigated by TALIS, it surveyed teachers’ perceptions about the degree of their control over the classroom issues. This variable was also assessed by a 4-point Likert scale ranging from 1 = strongly disagree to 4 = strongly agree. Five statements were used to collect teachers’ answers about their control over five areas regarding teaching and planning in the class (determine course content, select teaching methods, assess students’ learning, discipline students, and determine the amount of homework), the sample item is “How strongly do you agree or disagree that you have control over determining course content in the target class?”

Another mediating variable, *professional collaboration*, was measured by four items. These four items concentrated on the frequency of teachers’ engagement in collective activities, teamwork, group projects, and communication with colleagues (e.g., How often do you teach jointly as a team in the same class). Items were administered by teachers with the 6-point form (1 = never, 2 = once a year or less, 3 = 2–4 times a year, 4 = 5–10 times a year, 5 = 1–3 times a month, and 6 = once a week or more).

*Teacher innovativeness* was assessed with a 4-item 4-point scale ranging from 1 = strongly disagree to 4 = strongly agree. The items reflected how teachers felt held an open attitude and could develop new ideas or strategies for improving teaching and learning in the school (e.g., “Most teachers in this school strive to develop new ideas for teaching and learning.”).

Finally, teachers’ *gender* (0 = male, 1 = female), *educational level* (0 = below master, 1 = master and above), and *teaching experience in total* were added to the analysis as control variables regarding their possible influence on teacher innovativeness (Tang, 2021).

### Analytical approach

Firstly, out of the concern for Simpson’s paradox, this study initially detected the reliability and validity of each construct for each country/economy. Any country/economy that did not meet the acceptable standard was removed to ensure the appropriateness of combining the teacher data together. For reliability, this study did not use traditional Cronbach’s alpha since it requires strict assumptions, such as tau-equivalence, uncorrelated errors, and unidimensional scale, which cannot be satisfied in most studies. According to McNeish’s (2018) suggestion, Coefficient *H* is a proper alternative to Cronbach’s alpha. It does not require the tau-equivalence assumption for the scale and can take each item’s factor loading into consideration when calculating the optimally weighted reliability. Therefore, this study adopted Coefficient *H* to assess the reliability of the construct. Confirmatory factor analysis (CFA) was applied to determine the construct validity. The model fit indicators, including Chi-square (*χ*^2^), degrees of freedom (*df*), root mean square error of approximation (RMSEA), comparative fit index (CFI), Tucker-Lewis index (TLI), and standardized root mean square residual (SRMR), were evaluated. Meanwhile, factor loading for each item under the construct was also calculated. Secondly, after getting the final teacher data, descriptive analysis and correlation analysis were conducted for all constructs. Thirdly, four measurement models were evaluated using CFA based on the combined teacher data; model fit indicators mentioned above and factor loading were used to assess the acceptance of the measurement models. Lastly, the structural equation model (SEM) was estimated by maximum likelihood estimation (MLR) for exploring the structural relationships, including direct and indirect effects, between variables. The bootstrap method was applied to examine the mediation effect. Model fit indicators were also tested for the SEM. The standardized coefficients (*β*), standard error (SE), and coefficients of 95% confidence interval (CI) for direct and indirect effects were outputted. All the analyses were conducted in SPSS 25.0 and Mplus 8.3.

## Results

### Preliminary analysis

To begin with, this study examined the validity and reliability of each construct for each country/economy. In the validity test, based on the results of confirmatory factor analysis, four countries (Australia, Bulgaria, New Zealand, and Portugal) were excluded since they showed poor measurement model fit for the teacher autonomy construct and three countries/economies (Italy, Shanghai (China), and Spain) were excluded because they demonstrated unsatisfied construct validities for professional collaboration. In the reliability test, items’ factor loadings in each construct were used to calculate the Coefficient *H*; 10 countries (Belgium, Bulgaria, France, Norway, Portugal, Singapore, Slovak Republic, Slovenia, Sweden, and Vietnam) were excluded because of their low reliabilities for the professional collaboration construct (The results of reliabilities and validities of four constructs for each country/economy are available in the Supplementary Material). Finally, 15 countries/economies in total were excluded for further study, and 132,376 teachers from 33 countries/economies were used as the final sample in the following analysis.

### Descriptive analysis

Descriptive analysis was conducted using the final sample of 132,376 teachers from 33 countries or economies. The means and standard deviations of four variables are demonstrated in Table 1. The results indicated that teacher autonomy scored higher than the other three variables (*M* = 3.342, *SD* = 0.543). The mean of teacher innovativeness almost reached 3 (*M* = 2.994, *SD* = 0.641). The scores of distributed leadership and professional collaboration were relatively close but lowest among four variables (*M* = 2.893, *SD* = 0.568; *M* = 2.827, *SD* = 1.094).

**Table 1:** Descriptive analysis and correlation among variables.

	M	SD	DL	TA	PC	TI
DL	2.893	0.568	–			
TA	3.342	0.543	0.196^***^	–		
PC	2.827	1.094	0.360^***^	0.070^***^	–	
TI	2.994	0.641	0.457^***^	0.142^***^	0.349^***^	–

*** *p* < 0.001.

The correlation matrix in Table 1 implied that all variables are significantly correlated at the significance level of 0.001. Distributed leadership and teacher innovativeness showed the strongest correlation. However, the relationship between teacher autonomy and professional collaboration was the weakest.

### Measurement model

Before conducting the structural equation model, this study examined the measurement model for each construct based on confirmatory factor analysis using the combined teacher data.

The CFA results in Table 2 revealed model fit indicators for all constructs. Specifically, good model fit indicators were got for distributed leadership (*χ*^2^ = 30850.878, *df* = 5, RMSEA = 0.082, CFI = 0.908, TLI = 0.915, SRMR = 0.050), teacher autonomy (*χ*^2^ = 4766.869, *df* = 5, RMSEA = 0.085, CFI = 0.983, TLI = 0.965, SRMR = 0.022), professional collaboration (*χ*^2^ = 898.489, *df* = 2, RMSEA = 0.058, CFI = 0.989, TLI = 0.966, SRMR = 0.015), and teacher innovativeness (*χ*^2^ = 450.626, *df* = 2, RMSEA = 0.041, CFI = 0.999, TLI = 0.996, SRMR = 0.004). Taking one step further, the factor loading coefficients of items of distributed leadership, teacher autonomy, professional collaboration, and teacher innovativeness were discovered ranging from 0.711 to 0.800, from 0.619 to 0.832, from 0.511 to 0.680, and from 0.809 to 0.893. These two aspects of results indicated the measurement models were statistically acceptable for processing the structural equation model.

**Table 2:** Reliabilities and validities of constructs.

Construct	Items	Coefficient *H*	*χ* ^2^	*df*	RMSEA	CFI	TLI	SRMR
DL	5	0.876	30850.878	5	0.082	0.908	0.915	0.050
TA	5	0.871	4766.869	5	0.085	0.983	0.965	0.022
PC	4	0.698	898.489	2	0.058	0.989	0.966	0.015
TI	4	0.917	450.626	2	0.041	0.999	0.996	0.004

### Structural equation model

Model fit indicators suggested that the structural equation model had a satisfactory fit (*χ*^2^ = 57725.664, *df* = 181, RMSEA = 0.049, CFI = 0.948, TLI = 0.941, SRMR = 0.034).

**Figure 2:**
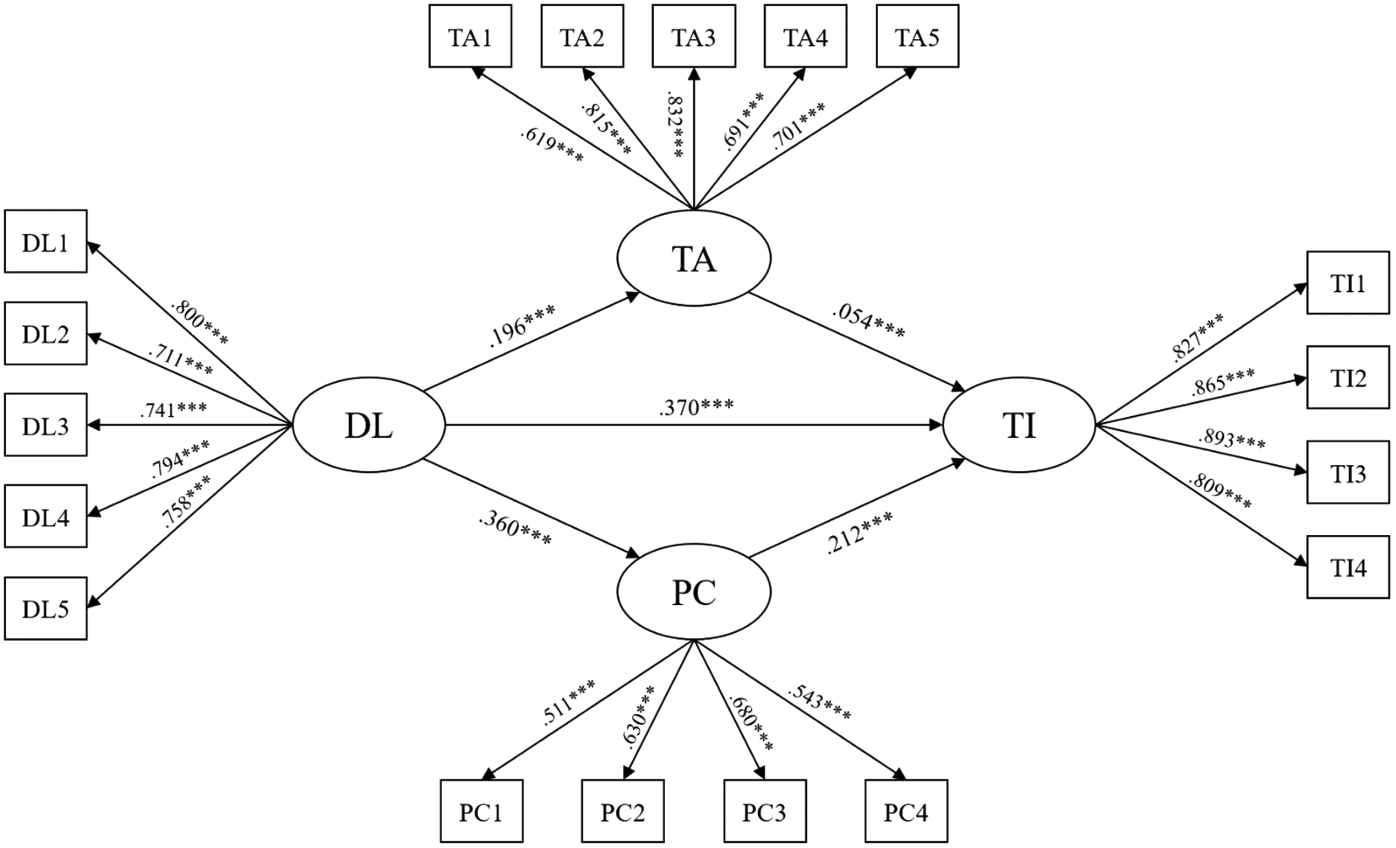
Structural equation model. ***p < 0.001.

To address the first research question, the study examined direct effects between variables. The results are illustrated in Table 3 and Figure 2. It showed that distributed leadership positively affected teacher innovativeness with the highest coefficient (*β* = 0.370, *p* < 0.001). Meanwhile, distributed leadership also significantly impacted teacher autonomy and professional collaboration (*β* = 0.196, *p* < 0.001; *β* = 0.360, *p* < 0.001). Although the coefficient was relatively small, it was also determined that when teachers owned more individual autonomy, they tended to report higher innovativeness (*β* = 0.054, *p* < 0.001). Similarly, teacher innovativeness was also significantly influenced by their engagement in professional collaboration activities (*β* = 0.212, *p* < 0.001).

**Table 3:** Structural equation modelling results with standardized coefficients.

Path	*β*	SE	95% CI	
			Lower	Upper
**Direct effect**				
DL → TA	0.196^***^	0.003	0.190	0.202
DL → PC	0.360^***^	0.003	0.353	0.366
DL → TI	0.370^***^	0.003	0.364	0.376
TA → TI	0.054^***^	0.003	0.049	0.060
PC → TI	0.212^***^	0.003	0.206	0.219
**Indirect effect**				
DL → TA → TI	0.011^***^	0.001	0.010	0.012
DL → PC → TI	0.076^***^	0.001	0.074	0.079

*** *p* < 0.001.

For the second research question, this study explored the indirect impacts of distributed leadership on teacher innovativeness through the mediation of teacher autonomy and professional collaboration. As Hayes (2009) noted, the indirect impact is statistically significant when “0” does not fall between the lower and upper boundaries of the 95% CI. According to Table 3, distributed leadership significantly affected teacher innovativeness through teacher autonomy with a small coefficient of the mediation effect (*β* = 0.011, *p* < 0.001). This indirect effect was also discovered in the relationship between distributed leadership, teacher innovativeness, and the mediation of professional collaboration (*β* = 0.076, *p* < 0.001). The total indirect effect of distributed leadership on teacher innovativeness was found statistically significant (*β* = 0.087; *p* < 0.001).

## Discussion and conclusion

The current study explored the effect of distributed leadership on teacher innovativeness using the teacher data derived from the 2018 wave of TALIS. This exploratory study applied a structural equation model to focus on the relationship between distributed leadership and teacher innovativeness, with a specific emphasis on the mediating roles of teacher autonomy and professional collaboration. The results of the study are discussed below.

Firstly, distributed leadership has positive direct effects on teacher innovativeness, teacher autonomy, and professional collaboration. The associations found between these factors are consistent with previous studies. Amels et al. (2020) discovered that teachers’ perceptions of distributed leadership might have a favorable influence on their motivation and capacity for school development and educational change. In two online e-learning projects in the United Kingdom, Jameson et al. (2006) concluded that distributed leadership effectively brought teachers together and encouraged teacher cooperation. Besides, a recent study also showed that distributed leadership of principals promoted teacher autonomy and teacher collaboration, respectively (Liu et al., 2021b). It is worth noting that the impact of distributed leadership on teacher innovativeness is more significant than its effects on the other two constructs, which indicates the importance of the participation of teachers in school decision-making for the creative attitudes and innovative actions of school employees. Buske (2018) also highlighted that in conjunction with the teaching staff’s perceptions of hierarchies inside the organization, the style of principals’ leadership is the most critical determinant of employees’ innovation capability.

Secondly, teacher autonomy positively affects teacher innovativeness and has a modest mediation effect in the influence of distributed leadership on teacher innovativeness. From an individual perspective, when teachers are given a sufficient amount of personal autonomy in the classroom, they are able to take innovative measures in their own classrooms to improve teaching and learning. Nguyen et al. (2021), who used data of teachers in 48 countries, concluded that teachers’ reported degrees of individual autonomy were a reliable indicator of teachers’ innovativeness. Nevertheless, the close relationship between three variables mentioned above also suggests that the leadership styles of principals not only directly affect the innovation capability of their staff but also enhance their creative behaviors by empowering individual teachers. It is also believed that when teachers regard their schools to be more collegial and supportive of teacher competency and autonomy, they tend to be more motivated and more eager to continue implementing educational innovation (Lam et al., 2010).

Thirdly, teachers’ professional collaboration positively impacts their innovativeness, and it also plays a potential mediating role between distributed leadership and teacher innovativeness. The tight correlation between professional collaboration and teacher innovativeness found in this study is consistent with several former pieces of research (Fullan, 2016; Torfing, 2019). Meanwhile, from a collective perspective, distributed leadership reduces the interpersonal distance between principals and individual teachers, and the school staff is then encouraged to initiate collaborative innovation together (de Jong et al., 2020). It is noteworthy that the direct effect of professional collaboration on teacher innovativeness is much stronger than the effect of teacher autonomy on innovativeness, and this phenomenon also exists in their mediation effects. One possible explanation is that when teachers operate in a professional community, they tend to share their expertise, knowledge, and creativity together. Consequently, the innovative capacity of a group of professionals is greater than that of an individual. Stuermer et al. (2009) also argued that innovativeness generated collectively is a different trait that surpasses the amount of innovativeness just at the individual level. Collective innovativeness may be characterized between a merely reductionist perspective and a holistic approach as an evolving phenomenon that is more powerful than personal innovativeness.

Implications for school leaders and individual teachers can be drawn based on our findings. School leaders, when focusing on promoting teacher innovativeness, are expected to adopt distributed leadership style, according to our results. Principals and school staff should establish a secure, friendly, and supportive school environment. In such contexts, teachers’ confidence in their ability to attain their aims should grow, and they are supposed to get more engaged in professional and collaborative work. Nevertheless, principals should also foster individual autonomy, assist teachers in performing creative work, and provide them with the opportunity to examine their situations and choose agendas that are appropriate to those circumstances (Buske, 2018). For individual teachers, it is expected to cultivate a culture of collectivism in their teaching profession by creating professional learning communities. It should become common for teachers to participate in various kinds of cooperative innovative practices, such as sharing feedback with one another regarding their innovation in instructional practices and taking part in collaborative professional development activities that take place on-site. In addition, teachers are expected to acquire the skills necessary to make effective use of the autonomy afforded to them in the classroom, particularly with regard to the development of new pedagogical practices and the integration of diverse forms of pedagogical content and technology. Compatibility across all of these aspects may further improve teachers’ innovativeness.

There are two limitations to this study. On the one hand, the teacher data derived from the 2018 TALIS were self-reported and assessed using a subjective Likert-type scale. Therefore, the concentration of the data was placed on the perceptions of teachers, which may not have been an accurate representation of the objective facts. On the other hand, the cross-sectional data in the TALIS were obtained simultaneously. As a result, it is hard to ascertain the causalities that exist among variables. For future studies, longitudinal investigations are anticipated to be carried out with the purpose of determining this.

Despite these limitations, the current study makes two contributions to the field of research. First, the study discovers generalized evidence about the influence of distributed leadership on teacher innovativeness in an international context, emphasizing the need for paying more attention to principals’ distributed leadership strategies when considering examining the related variables and influence mechanisms of school staff innovation. Second, evidence about the potential mediating roles of teacher autonomy and professional collaboration in the relationship between distributed leadership and teacher inventiveness was provided. Compared with previous studies, two unique working forms of teachers, individually and collectively, with distinct functions are newly determined and connected with their innovative behaviors and school leadership. In this way, the relationship between these constructs is further specified. Together, these contributions fill the research gaps and underline practical suggestions for fostering innovativeness among teachers. Looking forward, given that teacher innovativeness is a concept with worldwide significance and a necessary pre-requisite for the transformation of educational institutions into inventive organizations that cultivate the creative ability of teachers and students, it is imperative that more thorough empirical and conceptual studies pertinent to teacher innovation be explored in the future in order to build a strong knowledge foundation that can support innovative teaching and learning.

## Data availability statement

Publicly available datasets were analyzed in this study. This data can be found at: https://www.oecd.org/education/talis/talis-2018-data.htm.

## Ethics statement

The studies involving human participants were reviewed and approved by Organization for Economic Cooperation and Development (OECD). Written informed consent for participation was not required for this study in accordance with the national legislation and the institutional requirements.

## Author contributions

QL was in charge of this paper, made the research design, conducted data analysis, and wrote the whole section.

## Funding

This study was funded by China Social Science Foundation (Grant number: AFA210017).

## Conflict of interest

The author declares that the research was conducted in the absence of any commercial or financial relationships that could be construed as a potential conflict of interest.

## Publisher’s note

All claims expressed in this article are solely those of the authors and do not necessarily represent those of their affiliated organizations, or those of the publisher, the editors and the reviewers. Any product that may be evaluated in this article, or claim that may be made by its manufacturer, is not guaranteed or endorsed by the publisher.
